# How do clinical and socioeconomic factors impact on work disability in early axial spondyloarthritis? Five-year data from the DESIR cohort

**DOI:** 10.1093/rheumatology/keab607

**Published:** 2021-07-28

**Authors:** Elena Nikiphorou, Annelies Boonen, Bruno Fautrel, Pascal Richette, Robert Landewé, Désirée van der Heijde, Sofia Ramiro

**Affiliations:** 1 Department of Rheumatology, Leiden University Medical Center (LUMC), Leiden, Netherlands; 2 Centre for Rheumatic Diseases King's College London; 3 Department of Rheumatology, King’s College Hospital, London, UK; 4 Care and Public Health Research Institute (CAPHRI), Maastricht University, Maastricht; 5 Division of Rheumatology, Department of Internal Medicine, Maastricht University Medical Center, Maastricht, The Netherlands; 6 Department of Rheumatology, Pitie Salpetriere Hospital, Pierre Louis Institute for Epidemiology and Public Health, Sorbonne University—Assistance Publique Hopitaux de Paris, INSERM UMRS 1136, PEPITES Teams; 7 Department of Rheumatology, Hopital Lariboisière, Université de Paris, INSERM U1132, Paris, France; 8 Department of Rheumatology, Zuyderland Medical Center, Heerlen; 9 Department of Rheumatology & Clinical Immunology, Amsterdam University Medical Center, Amsterdam, Netherlands

**Keywords:** axSpA, work disability, disease activity, socioeconomic factors, adverse work outcomes

## Abstract

**Objectives:**

To investigate the impact of clinical and socioeconomic factors on work disability (WD) in early axial spondyloarthritis (axSpA).

**Methods:**

Patients from the DESIR cohort with a clinical diagnosis of axSpA were studied over 5 years. Time to WD and potential baseline and time-varying predictors were explored, with a focus on socioeconomic (including ethnicity, education, job-type, marital/parental status) and clinical (including disease activity, function, mobility) factors. Univariable analyses, collinearity and interaction tests guided subsequent multivariable time-varying Cox survival analyses.

**Results:**

From 704 patients eligible for this study, the estimated incidence of WD among those identified as at risk (*n* = 663, 94%), and across the five years of DESIR, was 0.05 (95% CI 0.03, 0.06) per 1000 person-days. Significant differences in baseline socioeconomic factors, including lower educational status and clinical measures, including worse disease activity, were seen in patients developing WD over follow-up, compared with those who never did. In the main multivariable model, educational status was no longer predictive of WD, whereas the AS disease activity score (ASDAS) and the BASFI were significantly and independently associated with a higher hazard of WD [HR (95%CI) 1.79 (1.27, 2.54) and 1.42 (1.22, 1.65), respectively].

**Conclusion:**

WD was an infrequent event in this early axSpA cohort. Nevertheless, clinical factors were among the strongest predictors of WD, over socioeconomic factors, with worse disease activity and function independently associated with a higher hazard of WD. Disease severity remains a strong predictor of adverse work outcome even in early disease, despite substantial advances in therapeutic strategies in axSpA.

Rheumatology key messages

- In early axSpA, clinical factors are amongst the strongest predictors of work disability.
- Worse disease activity and function independently associate with a higher hazard of work disability.
- Disease severity remains a strong predictor of work disability, despite substantial therapeutic advances.

## Introduction

Axial spondyloarthritis (axSpA) primarily affects people in their second or third decade of life, resulting in significant pain and disability [1–3]. Because the majority of people with axSpA are young and of working age, this makes work disability an important adverse outcome to study, which, besides its effect on the individual affected and people around them, also has wider societal impact [4, 5]. Reported work disability rates in axSpA have generally been consistently high across studies and countries [6–13]. Many studies to date, however, have focussed on established, radiographic (r-axSpA) disease. For example, French data suggest cumulative work withdrawal rates of 36% after 20 years of disease [7]; Finnish data report 5% withdrawal rates after 10 years, 30% after 25 years [8]; Spanish data report on permanent work disability in patients with mean (s.d.) disease duration 14 (10) years in r-axSpA of 26% [11]. Variations in the reported rates partly stem from country-level differences in national work disability schemes, social security and policy systems, but also from methodological differences across studies, such as differences in definitions used for work-related outcomes [14]. Furthermore, much of the literature to date reporting on loss of work in axSpA is out-dated. More recent data from the British Society for Rheumatology Biologics Register (BSRBR-AS), which recruited biologic naïve patients between 2012 and 2017, reported a very low rate for loss of employment, of 4.4% [including both r-axSpA and non-radiographic axSpA (nr-axSpA)] [15]. Further analyses on BSRBR-AS data suggest treatment with biologics to be associated with significantly greater improvements in presenteeism, work impairment and activity impairment with no differences seen in absenteeism, the strongest predictor of future work status such as work disability [16]. In this line, late-stage work outcomes including work status do not seem to be reversed by biological therapy alone [17], raising the question around a potential role of contextual factors that could be implicated in work disability [18]. This is particularly so in the biologics era and with the potential in recent times to diagnose and treat axSpA in the earlier stages of disease [19].

Although evidence to date suggests that worse disease activity and physical function as well as socioeconomic factors such as older age and lower social class are implicated in work disability in axSpA, much of the literature is based on cross-sectional data and/or established r-axSpA [13, 20–23]. The sparse data in early axSpA seem to suggest an impact of disease activity on adverse work outcomes, although data remain limited, also in terms of the length of follow-up studied. For example, data from the SpondyloArthritis Caught Early cohort, which included patients with early axSpA, demonstrate a striking impact of higher disease activity on total work impairment due to disease over one year [24].

The study of work disability and its associations in early axSpA remains poorly examined and represents an unmet need. This formed the rationale of this study, which used five-year data from the DESIR cohort to study work disability and potential factors associated with it, with a focus on both clinical and socioeconomic factors.

## Methods

### Study population

The study was based on the French DEvenir des Spondyloarthrites Indifférenciées Récentes (DESIR) cohort, which is a prospective, multicentre (*n* = 25) cohort (clinical trials.gov ID: NCT01648907) [25]. The cohort included consecutive patients with inflammatory back pain lasting ≥3 months but <3 years and with a clinical diagnosis of axSpA according to the treating rheumatologist. Patients who provided work-related data as part of the health-economic section of the DESIR Case Reporting Form (CRF) were included in the study. Both tick-box and free-text information provided in the CRF were used to maximize the information available related to work status and outcomes. Patients were grouped into one of two main categories: working or non-working, with relevant sub-categories for each.

### Dependent variable: work disability

Information on work disability was obtained via three main routes: (i) questions where the patients were asked to declare reasons for not working, with work disability included as an option; (ii) questions asking for a date of start of work disability; and (iii) free-text information provided by patients including reasons for not working. A patient was categorized as being work disabled if a date of disability was provided or if work disability was reported as a reason for not working. A separate study in DESIR with a focus on sick leave is currently under submission [26].

When a patient reported work disability as the reason for not working but did not provide a date in the relevant question on the CRF for work disability, the date of work disability was imputed as the mid-date between the current date of consultation (at which they reported being on work disability) and their previous date of consultation. This was the case for 33 observations (26 distinct patients) where the dates were imputed and included in the survival analysis (see below).

### Independent variables

The main independent variables included in the analyses were the socioeconomic and clinical variables, the latter focussing on disease activity, function and mobility variables. These were collected every 6 months in the first 2 years of follow-up, thereafter annually until 5 years.

#### Socioeconomic variables

The socioeconomic variables analysed where possible in their time-varying form included age, gender, educational status [low (primary or secondary education) *vs* high (university education)], ethnicity (Caucasian *vs* other), job type [blue-collar (manual labour work) *vs* white-collar (sedentary, office-based work)], marital status (married/in couple *vs* not) and parental status (number of children). Time-varying smoking status (current *vs* non-current smoker, since last visit) was also included in the analyses.

#### Clinical variables

Clinical variables were studied both in their baseline and time-varying form where available, to allow for changes in these variables over time and the effect on work disability, to be examined. For the main variables of interest, the AS disease activity score with CRP (ASDAS) and the BASDAI were used as measures of disease activity. Measures of physical function focussed on the BASFI and for spinal mobility, the BASMI.

Other clinical variables included in the analyses are described below. CRP measured in mg/l was used as the laboratory measure of systemic inflammation, in its continuous form, as well as in a binary form to indicate raised CRP (>6mg/l) at every visit. History of extra-muscular manifestations, namely uveitis, psoriasis and IBD were used in a binary form to indicate presence of these manifestations *vs* not, over time. Comorbidity burden was reflected in a comorbidity ‘count’ variable that was computed to include the following comorbidities: chronic pulmonary disease, ischaemic heart disease, pericarditis, heart failure, cardiac valve disease including aortic insufficiency, heart rhythm disorders, hypertension, cerebrovascular accidents, diabetes, gastric ulcers/perforation/haemorrhage, lymphoproliferative disease, organ neoplastic disease, suggestion of depression/anxiety using the Short Form-36 Mental Component Score (SF36_MCS; threshold of ≤38 to identify the presence of either depression or anxiety) [27]. The higher the count, the higher the number of comorbidities in an individual. Other disease characteristics recorded at entry into the study and used in their baseline form included: symptom duration studied as a continuous variable; presence of HLA-B27; and hip involvement.

Treatment variables were used in their time-varying form, namely: NSAIDs based on computation of the ASAS NSAID score (0–400) [28], also tested as binary variable (NSAID use in the last 6 months); conventional synthetic DMARD use; steroid use; and TNF-inhibitor use.

### Statistical analysis

Descriptive statistics for baseline characteristics of patients in the study population and separately for patients ‘at-risk’ of work disability among the total study population and at any point during follow-up were applied. Patients retired and those with work disability reported prior to their baseline date of consultation were considered as not ‘at-risk’ for work disability. The incidence rate of work disability was calculated based on the total observation time for those ‘at-risk’ for this outcome among the study population, in days, as all calculations of time were based on dates (with difference in days calculated between dates), for greater accuracy. The total at-risk observation time was calculated as the sum of the days that each patient was at risk from the time of entering the at-risk period to the end of follow up (5-year follow-up or earlier if lost to follow-up), censoring due to no longer at risk (retirement or work disability already at baseline) or to the event of interest, i.e. work disability (‘failure’).

Patients who at any point during the at-risk period developed work disability (‘Ever work disability’) were compared with those who did not report work disability during the at-risk observation period (‘Never work disability’) using the Wilcoxon test for continuous variables and either the χ^2^ or Fisher's exact test (applied for small samples) for categorical variables. Time-varying Cox survival analysis was used to study time to work disability for all patients who were at risk of work disability at any time-point during follow-up.

Firstly, interactions were tested between disease activity (ASDAS) or function (BASFI) and each of the following: age, gender and education; if statistically significant (*P* < 0.15) interactions were identified, model stratification was considered, if the interaction was also considered clinically relevant. Following this, univariable analysis was undertaken with work disability as the dependent outcome. Variables with a *P* <0.20 were subsequently tested in multivariable analyses. The latter was based on stepwise forward Cox regression model building, with independent variables retained in the models if significant at the *P* <0.05 level or if identified as confounders [resulting in a change of the hazard ratio (HR) of socioeconomic variables by >15%]. Because the focus of the analyses was on both socioeconomic factors as well as clinical variables, for the latter, separate models were built to explore the role of clinical factors that were significant univariably but not retained in a multivariable model with other clinical factors. This was to allow for the effects of these clinical factors to be clearly seen. Collinearity cheques were undertaken between individual variables, followed by testing of the final models for multi-collinearity and violation of proportional hazards. Finally, sensitivity analyses were performed in the subgroup fulfilling the Assessment of SpondyloArthritis international Society (ASAS) classification criteria for axSpA [29] following each of the above analysis steps.

### Ethics

The DESIR study was conducted according to good clinical practice guidelines and was approved by the appropriate local medical ethical committees (Comité lde Protection des Personnes Ile de France III) on 17 September 2007 (EUDRACT number: 2007-A00608-45). A detailed description of the study protocol is available at the DESIR website (www.lacohortedesir.fr/desir-in-english/). The research proposal for this particular analysis was approved by the scientific committee of the DESIR cohort.

## Results

Baseline characteristics of the study population (*n* = 704) are included in supplementary material (Supplementary Table S1, available at *Rheumatology* online). The cohort primarily consisted of young [mean (s.d.) age of 33.8 (8.6)], female (54%), Caucasian (90%) patients. The majority (80%) declared working upon entry into the cohort with 17% of these reporting a blue-collar profession.

At baseline, 1% reported being work disabled. Comparisons of baseline characteristics of people work disabled at baseline compared with those not disabled suggest significantly worse disease activity, functional ability and mobility in the former group, as well as lower educational status (Supplementary Table S2, available at *Rheumatology* online).

Ninety-four percent (*n* = 663) of the study population were ‘at-risk’ of work disability over the study duration and were included in the time-to-event analyses. In total, 46 (7%) patients developed work disability during the 5 years of follow-up. Mean (s.d.) time to work disability was 139 weeks (s.d. 68) (min 23, max 289 weeks). In people who developed work disability, 25% did so by 85 weeks; 50% and 75% by 150 and 205 weeks, respectively. The estimated incidence of work disability among those at risk and across the five years was 0.05 (95% CI 0.03, 0.06) per 1000 days, based on a total person-days of observation of 999 999 days.

Significant differences were noted in the baseline characteristics of patients at risk who reported work disability at any point during the study (‘Ever work disability’) compared with those who never did (‘Never work disability’). This was notable for socioeconomic factors where, for example, older age and lower education were seen in those who were ‘Ever’ on work disability (Table 1). In terms of biologic treatment, while there were no patients using them at the time of recruitment into DESIR, by year 5 there were 40% on a TNFi, accounting for those remaining in follow-up.

**Table 1:** Baseline characteristics of patients at risk of, and with ‘Ever’ and ‘Never’ work disability and for those with ‘Ever’ and ‘Never’ work disability during the study follow-up

	At risk of work disability	Ever work disability	Never work disability	*P* value^*^
Baseline variable	Total *n* = 663	Total *n* = 46	Total *n* = 617	
Age, years	33.7 (8.6)	39.5 (8.2)	33.3 (8.5)	<0.001
Male gender	306, 46%	17, 37%	289, 47%	0.195
Caucasian ethnicity	599, 90%	41, 89%	558, 90%	0.772
Higher education^a^	401, 61%	14, 30%	387, 63%	<0.001
Blue-collar profession^d^	95, 17%	11, 26%	84, 16%	0.064
Married/In couple^a^	424, 64%	36, 78%	388, 63%	0.04
Parental status, number of children^b^	1.1 (1.2)	1.6 (1.2)	1.1 (1.2)	0.002
Smoking, current^a^	235, 36%	20, 46%	215, 35%	0.160
HLA-B27 positivity^a^	384, 58%	23, 50%	361, 59%	0.254
Symptom duration, years^a^	1.5 (0.9)	1.4 (0.8)	1.5 (0.9)	0.235
ASDAS-CRP^b^	2.64 (0.9)	3.1 (0.9)	2.6 (0.9)	0.001
Elevated CRP (>6mg/L)^b^	179, 28%	11, 25%	168, 28%	0.654
CRP, mg/L^b^	7.75 (13.5)	8.9 (21.2)	7.7 (12.8)	0.247
BASDAI, 0–10^a^	4.45 (2.01)	5.9 (1.7)	4.3 (2.0)	<0.001
BASFI, 0–10^a^	3.01 (2.28)	5.2 (2.1)	2.8 (2.2)	<0.001
BASMI, 0–10^c^	2.43 (0.95)	3.2 (1.1)	2.4 (0.9)	<0.001
History of uveitis	58, 9%	1, 2%	57, 9%	0.102
History of psoriasis	114, 17%	8, 17%	106, 17%	0.971
History of IBD	34, 5%	2, 4%	32, 5%	0.804
History of peripheral arthritis^a^	46, 7%	5, 11%	41, 7%	0.278
Comorbidity count, 0–4^b^	0.6 (0.7)	1.0 (0.8)	0.6 (0.6)	<0.001
NSAID score in last week, 0–400^b^	56.4 (52.6)	69.5 (53.8)	55.4 (52.5)	0.099
TNFi use	0,0%	0,0%	0,0%	—
Steroid use	81, 12%	10, 22%	71, 12%	0.041
csDMARD use^a^	86, 13%	11, 24%	75, 12%	0.022

Mean (s.d.) for continuous variables; n, % for categorical variables. N number reported where there were missing data for the specific variable. Missing data: ^a^<1% missing; ^b^<5% missing; ^c^<10% missing; ^d^<15% missing. Comparisons between the ‘Ever sick leave’ *vs* the ‘Never sick leave’ categories were undertaken using the Wilcoxon test for continuous variables and either the χ^2^ or Fisher's exact test for categorical variables. ASDAS: AS disease activity score; csDMARD: conventional synthetic DMARD; TNFi: TNF inhibitor.

### Effect of socioeconomic and clinical factors on work disability

No clinically relevant interactions were identified between socioeconomic and clinical factors, so analyses were not stratified. Clinical variables, namely ASDAS, BASFI and BASMI were strongly significant univariably. However, the most parsimonious multivariable models could not retain all these variables. To see their individual effect, different models were built, with ASDAS featuring in the main model (also with the best statistical fit) along with BASFI, separately from a model whereby BASMI was retained, along with BASFI, but not ASDAS. BASFI was thus significantly predictive of work disability in both the ASDAS and the BASMI models, as shown in Table 2. In the ASDAS-based model, every unit increase in ASDAS was associated with a 79% increase in the hazard of work disability and every unit increase in BASFI increased the hazard of work disability by 42%. Education was retained in both models; in the ASDAS-based model its significance was lost upon addition of ASDAS with a relevant change in the HR of education by ∼30%, whereas in the BASMI model, education was an independent predictor, with high education decreasing the hazard of WD by 58%. Table 2 shows the final models, along with the explanatory variables tested in each of the models, based on the univariable findings.

**Table 2:** Effect of socio-economic and clinical variables on time to work disability, univariable analysis and separate multivariable models for ASDAS (main model) and BASMI

Type of analysis	Univariable	Multivariable model	Multivariable model
		Focus on ASDAS	Focus on BASMI
	HR (95% CI)	HR (95% CI)	HR (95% CI)
		(*n* = 653)	(*n* = 639)
Explanatory variables			
Age, years^***^	1.07 (1.04, 1.11)	1.06 (1.02, 1.10)	1.03 (0.99, 1.08)
Male gender	0.68 (0.37, 1.23)	1.10 (0.58, 2.08)	1.00 (0.50, 2.02)
High education^***^	0.25 (0.14, 0.48)	0.57 (0.29, 1.11)	0.42 (0.19, 0.90)
Parental status^**^	1.36 (1.09, 1.70)	NS	NS
ASDAS^***^	2.40 (1.77, 3.26)	1.79 (1.27, 2.54)	Not tested
BASFI, 0–10^***^	1.54 (1.36, 1.75)	1.42 (1.22, 1.65)	1.53 (1.29, 1.81)
BASMI, 0–10^***^	2.13 (1.65, 2.75)	NS	1.49 (1.10, 2.00)
Symptom duration, years^*^	0.79 (0.56, 1.12)	NS	NS
HLA B27 positive^*^	0.64 (0.36, 1.13)	NS	NS
Hip involvement (baseline)^*^	1.98 (1.02, 3.82)	NS	NS
Comorbidity count (0–4)^***^	2.33 (1.68, 3.21)	NS	NS
NSAID use last 6 months^*^	1.01 (1.00, 1.02)	NS	NS
Oral corticosteroid use^**^	2.72 (1.27, 5.83)	NS	NS
csDMARD use last 6 months^*^	2.20 (0.90, 5.41)	NS	NS
TNFi use^*^	2.15 (1.19, 3.87)	NS	NS

Number of ‘failures’ in the main (ASDAS) model = 42 and in the BASMI model = 34. *P <0.2; **P <0.05; ***P <0.005. ASDAS: AS disease activity score; csDMARD: conventional synthetic DMARD; NS: not significant; TNFi: TNF inhibitor.

### Subgroup analyses in patients fulfilling the ASAS classification criteria

In sensitivity analyses of the ASAS criteria fulfilling subgroup (*n* = 423, 60%), similar findings were observed with disease activity, whereby ASDAS was retained as a significant predictor of work disability [HR (95% CI) 1.88 (1.11, 3.18)] alongside physical function [HR (95% CI) 1.39 (1.11, 1.74)] in the most parsimonious model adjusting for age, gender and educational status (Table 3). Unlike the main study population model, however, high education in the model of the ASAS criteria fulfilling group, was significantly predictive of a lower hazard of disability [HR (95% CI) 0.32 (0.11, 0.95)].

**Table 3:** Effect of socio-economic and clinical variables on time to work disability in patients fulfilling the ASAS criteria

	Work disability
	HR (95% CI) *n* = 394
Explanatory variables	
Age (years)	1.08 (1.01, 1.15)
Male gender	0.72 (0.27, 1.91)
Higher education	0.32 (0.11, 0.95)
ASDAS	1.88 (1.11, 3.18)
BASFI, 0-10	1.39 (1.11, 1.74)
Model information	
Number of events (‘failures’)	19

ASDAS: AS disease activity score.

## Discussion

In this study of young, early axSpA individuals, with an average age of 34 at entry into the study and the majority working, work disability was a relatively infrequent phenomenon, as supported also by previous longitudinal data in axSpA [15]. The estimated incidence of work disability among those at risk and over the five years of follow-up was 0.05 (95% CI 0.03, 0.06) per 1000 days based on almost 1 million total person-days of observation. Across the study group, patients who developed work disability compared with those who never did were of significantly older age and lower education from a socioeconomic perspective and had worse disease markers, at entry into the study. Importantly, the effect of clinical variables, namely time-varying disease activity (ASDAS), functional ability (BASFI) and spinal mobility (BASMI) indicators persisted in (separate) multivariable models as independent predictors of work disability. Worse functional ability (BASFI) in particular featured in both models as a significant predictor of work disability, highlighting the relevance of this variable on this adverse work outcome, already in early disease and supporting previous literature from established r-axSpA [23, 30, 31].

The low rate of work disability in this young cohort of early axSpA patients may be a reflection of the earlier diagnosis and hence earlier and better management of disease, as well as the younger age of patients. This is an interesting and novel observation, compared with the substantial work disability reported already at diagnosis and high further rates of work disability based on previous (mainly cross-sectional) studies in long-term disease [7–11, 32]. This is an important finding and advocates the importance of early diagnosis and treatment, to potentially prevent work disability in axSpA. Whereas there were no patients on a TNFi at baseline, this number increased to 43% of patients in DESIR by year five. The use of TNFi over time was significant only univariably in our study. The absence of selection of this variable in the multivariable models, with the clinical variables predominating, suggests confounding by indication.

In this study and compared with previous analyses in DESIR with sick leave as an adverse work outcome [26], we observe that clinical variables seem to be of higher relevance when it comes to work disability, whereas contextual, socioeconomic factors appear to be more relevant to sick leave. Previous work from our group has shown lower education, older age and female gender to be independently associated with a higher hazard of sick leave in early axSpA. It may not be surprising that clinical factors play a stronger role in work disability, as the latter represents a more permanent consequence of disease and one likely more influenced by clinical indicators than by context. On the other hand, sick leave represents an outcome that is to a large extent an individual’s decision, aside from a disease-driven outcome and also a non-permanent work outcome. In this sense, contextual factors become potentially more relevant. It is also possible that socioeconomic factors including lower social class as reflected by lower educational level may gain more relevance in long-standing axSpA, explaining the predictive value of these contextual factors on work disability shown in previous studies on established disease [13]. In the case of education, an important clinical message is that rheumatologists should pay attention to patients with poorer educational backgrounds, keeping in mind that they may struggle to work or find alternative jobs, with relevant impact on their social circumstances, their condition and life as a whole. In some countries, including France, people can benefit from dedicated programs that aim to support them in completing their education and helping them to find other, more suitable occupations.

In this study on work disability, although education was significantly predictive of a higher hazard of this adverse work outcome in the BASMI model, it was not significant in the ASDAS model, although ASDAS confounded the effect of education on work disability. In the subgroup analyses of patients fulfilling the ASAS criteria, higher education was found to independently associate with a lower hazard work of disability in a model, again retaining both disease activity and physical function as significant predictors. Interestingly, type of job based on the collar categorization was not significantly associated with work disability. This may be a reflection of the inherent challenges of a rather crude categorization system based on type of job (manual/physically ‘demanding’ *vs* sedentary types of jobs) and which further prevents the study of the possible impact psychosocial requirements of specific jobs have on people.

We found increasing age to be associated with a higher hazard for work disability. Older age has been reported in previous studies as a predictor of work disability in r-axSpA [13], which may not be unexpected. Yet, in early axSpA, it is interesting to see the effect of age, even in this young group of patients. Of those who became work disabled, 50% did so before the third year (1050 days) from entry into the study. Both disease activity as measured by ASDAS and function (BASFI) were associated with a higher hazard of work disability in the most parsimonious model. For every unit increase in ASDAS, the hazard of work disability increased by 79% and for every unit increase in BASFI, the hazard of work disability increased by 42%. Previous studies, although most at cross-sectional level, have shown associations between work productivity impairment and higher disease activity and worse function in axSpA [23, 30, 31]. Longitudinal data in established r-axSpA based on the Outcome Assessments in Ankylosing Spondylitis International Study support these observations [33]. Furthermore, impairment in spinal mobility contributed to explain work disability, and independently from function, even in this cohort of patients with early axSpA. This emphasizes that clinical measures are relevant and predictive for adverse work outcomes. Recent data from the DESIR cohort looking at disease activity trajectories in early axSpA, albeit with a different methodology and research question and less follow-up, confirm the link between persistent higher disease activity in the first three years and work disability over time [34]. Our study goes a step further to provide an in-depth exploration of both clinical and socioeconomic time-varying factors over five years of follow-up, to understand the effect on work disability in early axSpA.

Limitations of our study include the lack of precision in some of the self-reported data on work disability; for example, the inconsistencies with dates *vs* tick-box responses provided to indicate work disability, necessitating data imputation where there was missing data. The lack of a direct question on the CRF asking patients specifically about their current work status and needing to derive this information from other less direct questions is a weakness. Additionally, for those patients included in the survival analyses and who were not at work at baseline [but were still ‘at risk’ as they could (and some did) later become employed], if their unemployment was related to their diagnosis, then this could bias our work disability estimates. However, there was no clear way to determine the cause of unemployment. Overall, a conservative approach was undertaken to ensure maximal and accurate use of all sources of information available and related to work disability. Analyses were focussed on time to first work disability, treating the latter as a permanent adverse work state. Another limitation was the low number of events (work disability) in this early axSpA cohort, which poses challenges – for example, due to power issues – when studying potential associations. Furthermore, and as outlined higher up, the categorization of jobs based on type of collar is perceived as rather crude and lacking appropriate detail for a deeper understanding of the impact of the physical and psychosocial aspects of different jobs. Finally, comorbidity burden was analysed in this study using a simple count of comorbidities that included both physical and mental health comorbidity. This approach could have prevented more direct associations to be seen between individual types of comorbidities and work disability. However, the latter would limit the analyses in view of the low numbers, and it was also beyond the scope of this study. We therefore opted for a simpler albeit ‘cruder’ way to study comorbidity burden and its impact, based on simple count.

Despite these limitations, our study also has several strengths. Firstly, it is one of only a few to examine work disability in *early* axSpA and with a respectable length of follow-up, enabling the study of time-varying covariates and making maximal use of the information on each patient, over time. The real-life, prospective data collection in DESIR and the large patient sample are additional strengths. The possibility to study socioeconomic factors gives a further dimension to analyses presented to date and provides more insights that complement the existing body of evidence.

In conclusion, our study provides additional evidence for the role of clinical and socioeconomic factors on work disability in early axSpA. The study demonstrates that despite substantial advances in the therapeutic strategies in SpA, disease severity as reflected particularly by high disease activity and poor function, as well as poor spinal mobility, remains a strong predictor of work disability. The findings suggest that addressing clinical indicators of worse disease through traditional monitoring and treat-to-target interventions is important also in supporting people living with axSpA to remain at work. At the same time, the low rates of work disability observed in this cohort of young, early axSpA patients, unlike previous studies in long-term disease, suggest a window of opportunity to intervene and prevent work disability in axSpA, through early diagnosis and management.

## Supplementary Material

keab607_Supplementary_Data
