# Supplementary material for: How do clinical and socioeconomic factors impact on work disability in early axial spondyloarthritis? Five-year data from the DESIR cohort
Source: Rheumatology (Oxford). 2021 Jul 28;61(5):2034–42. doi: 10.1093/rheumatology/keab607 (PMC9071517; doi:10.1093/rheumatology/keab607)
Supplement: keab607_Supplementary_Data [file keab607_supplementary_data.docx]

**Supplementary File**

**Supplementary Table S1.** Baseline characteristics of the study population and the subgroup fulfilling ASAS criteria.

| **BASELINE VARIABLES** | **Study population**  **N=704**  **Mean (SD) or n, %** | **ASAS criteria subgroup**  **N=423**  **Mean (SD) or n, %** |
| --- | --- | --- |
| Age, years | 33.8 (8.6) | 31.5 (7.3) |
| Male gender | 324, 46% | 223, 53% |
| Caucasian ethnicity | 631, 90% | 378, 89% |
| Higher education^ | 417, 59.4% | 270, 64% |
| Working | 561, 80% | 328, 78% |
| Blue-collar profession (of those working)^^^^ | 96, 17% | 64, 20% |
| Married/In couple^ | 445, 63% | 257, 61% |
| Parental status, number of children^^ | 1.1 (1.2) | 0.9 (1.1) |
| Smoking, current^ | 256, 37% | 167, 40% |
| HLA-B27 positivity^ | 409, 58% | 371, 88% |
| Symptom duration, years^ | 1.5 (0.9) | 1.6 (0.9) |
| ASDAS^^ | 2.7 (0.9) | 2.6 (1.0) |
| Elevated CRP (>6mg/L)^^ | 194 (28.5) | 135, 33% |
| CRP, mg/L^^ | 7.9 (13.6) | 8.7 (13.9) |
| BASDAI, 0-10^ | 4.5 (2.0) | 4.2 (2.0) |
| BASFI, 0-10^ | 3.0 (2.3) | 2.9 (2.2) |
| BASMI, 0-10^^^ | 2.4 (1.0) | 2.4 (0.9) |
| History of uveitis | 65, 9% | 43, 10% |
| History of psoriasis | 117, 17% | 63, 15% |
| History of IBD | 35, 5% | 20, 5% |
| History of peripheral arthritis^ | 49, 7% | 28, 7% |
| NSAID score in last week, 0-400^^ | 55.9 (52.6) | 61.6 (52.1) |
| TNFi use | 0, 0% | 0, 0% |
| Steroid use | 85, 12% | 59, 14% |

*ASDAS, Ankylosing spondylitis (AS) disease activity score; CRP, C-Reactive Protein; BASDAI, Bath AS disease activity index; BASFI, Bath AS functional index; BASMI, Bath AS Metrology Index; IBD, inflammatory bowel disease; NSAIDs, non-steroidal anti-inflammatory drugs; TNFi, Tumour Necrosis Factor inhibitor. *Indicates variable n where total n not available due to missing data. Missing data: ^<1% missing; ^^<5% missing; ^^^<10% missing; ^^^^<15% missing.*

**Supplementary Table S2.** Comparison of baseline characteristics between patients with work disability compared to those without, at entry into the cohort.

|  | **TIME: BASELINE** | | | | | | | | | | | | | |  |
| --- | --- | --- | --- | --- | --- | --- | --- | --- | --- | --- | --- | --- | --- | --- | --- |
|  | **No Disability (n=697)** | | | | | | | **Disability (n=7)** | | | | | | |  |
|  | **N** | **mean** | **SD** | **p50** | **IQR** | **min** | **max** | **N** | **Mean** | **SD** | **p50** | **IQR** | **min** | **max** | **p-value** |
| **CRP, mg/L** | 675 | 7.9 | 13.6 | 3.1 | 5.1 | 0.1 | 126.3 | 6 | 7.0 | 5.5 | 6.0 | 4 | 1.1 | 17.1 | 0.268 |
| **ASDAS** | 669 | 2.6 | 0.9 | 2.6 | 1.4 | 0.6 | 6.2 | 6 | 3.1 | 0.5 | 3.1 | 0.7 | 2.4 | 3.7 | 0.155 |
| **BASDAI, 0-10** | 695 | 4.5 | 2.0 | 4.5 | 3.1 | 0 | 10 | 7 | 6.0 | 1.2 | 6.2 | 1.3 | 3.5 | 7.3 | 0.026 |
| **BASFI, 0-10** | 692 | 3.0 | 2.3 | 2.5 | 3.6 | 0 | 9.8 | 7 | 5.2 | 1.8 | 5.9 | 1.6 | 1.4 | 6.5 | 0.013 |
| **BASMI,0-10** | 634 | 2.4 | 1.0 | 2.3 | 1.2 | 0.3 | 6.1 | 6 | 3.5 | 1.2 | 3.5 | 1.1 | 1.6 | 5.1 | 0.028 |
| **NSAID score in past week** | 683 | 56.3 | 52.7 | 50 | 100 | 0 | 200 | 7 | 16.3 | 23.5 | 1.3 | 50 | 0 | 50 | 0.062 |
| **Comorbidity count, 0-4** | 687 | 0.6 | 0.7 | 1 | 1 | 0 | 4 | 7 | 0.9 | 0.4 | 1 | 0 | 0 | 1 | 0.150 |
| **Parental status** | 677 | 1.1 | 1.2 | 1 | 2 | 0 | 6 | 7 | 1.4 | 1.3 | 1 | 3 | 0 | 6 | 0.423 |
|  | **Frequency** | | | **Percent*** | | | | **Frequency** | | | **Percent*** | | | | **p-value** |
| **Caucasian ethnicity** | 625 | | | 89.7 | | | | 6 | | | 85.7 | | | | 0.537 |
| **Higher Education** | 416 | | | 59.9 | | | | 1 | | | 14.3 | | | | 0.020 |
| **Married/In couple** | 442 | | | 63.6 | | | | 3 | | | 42.9 | | | | 0.267 |
| **Blue-collar profession** | 104 | | | 17.2 | | | | 1 | | | 25.0 | | | | 0.532 |
| **Smoking, current** | 251 | | | 36.3 | | | | 5 | | | 71.4 | | | | 0.107 |
| **History of uveitis** | 62 | | | 8.9 | | | | 3 | | | 42.9 | | | | 0.020 |
| **History of psoriasis** | 117 | | | 16.8 | | | | 0 | | | 0.0 | | | | 0.607 |
| **History of IBD** | 34 | | | 4.9 | | | | 1 | | | 14.3 | | | | 0.301 |
| **History of peripheral arthritis** | 48 | | | 6.9 | | | | 1 | | | 14.3 | | | | 0.398 |
| **Steroid use** | 85 | | | 12.2 | | | | 0 | | | 0.0 | | | | 1.000 |
| **TNFi use** | 0 | | | 0.0 | | | | 0 | | | 0.0 | | | | - |
| *% of non-missing values | | | | | | | | | | | | | | | |

*ASDAS, Ankylosing spondylitis (AS) disease activity score; CRP, C-Reactive Protein; BASDAI, Bath AS disease activity index; BASFI, Bath AS functional index; BASMI, Bath AS Metrology Index; IBD, inflammatory bowel disease; NSAIDs, non-steroidal anti-inflammatory drugs; TNFi, Tumour Necrosis Factor inhibitor. P-values: The Wilcoxon test used to compare continuous variables and the Fisher's exact test for categorical variables.*

*.*
